# Central Nervous System and Peripheral Expression of CCL19, CCL21 and Their Receptor CCR7 in Experimental Model of Multiple Sclerosis

**DOI:** 10.1007/s00005-015-0339-9

**Published:** 2015-05-10

**Authors:** Bartosz Bielecki, Izabela Jatczak-Pawlik, Pawel Wolinski, Andrzej Bednarek, Andrzej Glabinski

**Affiliations:** Department of Neurology and Stroke, Medical University of Lodz, Lodz, Poland; Department of Propedeutics of Neurology, Medical University of Lodz, Lodz, Poland; Department of Molecular Cancerogenesis, Medical University of Lodz, Lodz, Poland; U1195 Inserm and University Paris-Sud, 80 General Leclerc st., 94276 Le Kremlin-Bicetre, France

**Keywords:** Experimental autoimmune encephalomyelitis, Chemokine, Chemokine receptor, Neuroinflammation

## Abstract

It is well documented that inflammatory chemokines play a significant role in the development of multiple sclerosis (MS) and its model, experimental autoimmune encephalomyelitis (EAE). Recently, the involvement of homeostatic (or lymphoid) chemokines in the pathogenesis of autoimmune diseases has become an object of intensive study. In this work, quantitative analysis of CCL19, CCL21 and CCR7 expression in the central nervous system (CNS), as well as in inflammatory mononuclear cells isolated from several organs during the first attack, remission and the second attack of chronic-relapsing EAE (ChREAE), was performed. Using real-time PCR, RNAse Protection Assay and immunohistochemistry, the expression of both chemokines, as well as of their common receptor CCR7, was analyzed in the brain, spleen, lymph nodes and peripheral blood mononuclear cells. Increased expression of CCL19 and CCL21 was observed mostly in mononuclear inflammatory cells isolated from the CNS during active ChREAE. At the same time the expression of CCR7 in blood mononuclear leukocytes was reduced. This observation extends our current knowledge about the possible role of chemokines CCL19, CCL21 and their receptor CCR7 in the pathogenesis of ChREAE and, by extension, MS.

## Introduction

Multiple sclerosis (MS) is a disease of the central nervous system (CNS) affecting predominantly young adults. It is characterized by a diverse clinical picture and a complex physiopathology. Main histopathological hallmarks of the disease include myelin damage and axonal loss, which are believed to be a consequence of autoimmune inflammation (Frohman et al. 2006; Lassmann et al. 2001). In order to better understand the immunopathology of MS, various experimental models have been established with the most renowned being experimental autoimmune encephalomyelitis (EAE). A clinical resemblance to the most common form of MS, relapsing-remitting MS, makes chronic-relapsing type of EAE (ChREAE) a particularly attractive model. It helps to study the sequence of events leading to a development of neuroinflammation as well as the relation between clinical signs and reactivation of CNS inflammation. It has been shown that the clinical course of EAE correlates well with changes of expression of proinflammatory and immunomodulatory cytokines and chemokines in the brain and spinal cord (Glabinski et al. 2003b).

We know from earlier studies that the inflammatory chemokines CXCL10 and CCL2 are upregulated during acute EAE in the brains of mice (Ransohoff et al. 1993) and rats (Hulkower et al. 1993).

Recently, also homeostatic (or lymphoid) chemokines and their receptors have become the object of intensive research. They are known to play a pivotal role in physiological processes but also in pathological conditions such as autoimmune inflammation.

The common receptor for both CCL19 and CCL21 is CCR7, which is expressed in dendritic cells (DCs) and T-cells. CXCR3, a primary receptor for chemokines CXCL9 and CXCL10, is also known to be activated by chemokine CCL21 (Rappert et al. 2002). Previously, we observed that during ChREAE the expression of CCR7 increases in the brain and spinal cord and it is limited to the inflammatory cells infiltrating the CNS parenchyma (Bielecki et al. 2007). Increased levels of CCL19 and CCL21 have also been shown in the cerebrospinal fluid of patients with MS-type optic neuritis and patients with other inflammatory neurological diseases (Pashenkov et al. 2003). This suggests that lymphoid chemokines can also play an important role in the development and regulation of CNS inflammation.

Despite intensive study, the involvement of CCR7 and its ligands CCL19 and CCL21 in the process of cell migration and the formation of inflammatory lesions in the CNS is still not fully understood. The main goal of this study was to analyze thoroughly the expression of CCL19, CCL21 and CCR7 during different stages of ChREAE. A quantitative study of CCL19, CCL21 and CCR7 was performed during the first attack, remission, and the second attack of ChREAE. The organs of interest were the brain, spleen, lymph nodes and blood. The main objective was to measure the expression of the chemokines of interest in inflammatory cells during early stages of the disease. In order to achieve this we isolated mononuclear cells from the brain, spleen and lymph nodes, as well as the peripheral blood mononuclear cells (PBMCs) from blood. In the following part of the study, we confirmed which cell types were the source of CCL19 and CCL21 in the CNS during ChREAE in the CNS.

## Materials and Methods

### Animals

In all experiments 8–12-week-old female (SWR × SJL) F1 mice were used. They were the first generation of SWR/J (H-2q) female and SJL/J (H-2s) male mice obtained from The Jackson Laboratory (Bar Harbor, ME, USA). All animals were housed at the animal facility of The Medical University of Lodz (Lodz, Poland) under standard conditions. All experimental protocols were approved by the Animal Care Committee of The Medical University of Lodz.

### Induction of ChREAE

ChREAE was induced by subcutaneous injection of 139–151 PLP (proteolipid protein peptide: HSLGKWLGHPDKF) emulsified with complete Freund’s adjuvant (Sigma-Aldrich, Poland). Additionally, Pertussis toxin (Sigma-Aldrich, Poland) was injected intravenously at the day of immunization and 72 h later, as previously described (Glabinski et al. 1997b). The induction rate was above 95 %. Animals were weighed and examined daily. Average disease onset was observed between day 10 and 14 after immunization. Severity of the disease was evaluated using a clinical scoring scale as follows: grade 0—no clinical symptoms; grade 1—decreased tail tone or slightly clumsy gait; grade 2—tail atony and/or moderately clumsy gait and/or poor righting ability or cerebellar ataxia; grade 3—hind limb weakness; grade 4—hind limb paralysis or four limb weakness; grade 5—tetraplegia or moribund state. First attack and relapse onset were defined as the day when the first or the new (respectively) clinical signs of the disease appeared. Onset of remission was defined as the day when clinical signs improved (Glabinski et al. 1997a). Naïve (non-immunized) animals matching immunized mice served as a negative control and were sacrificed at 10 weeks of age.

### Extraction of RNA from Tissue Homogenate

At predetermined time points after the disease onset mice were anesthetized with a ketamine/xylazine (Biowet, Poland) cocktail (100 mg/kg of ketamine and 10 mg/kg of xylazine) and perfused through the left cardiac ventricle with ice cold phosphate-buffered saline (PBS; Sigma-Aldrich, Poland) with heparin. The organs selected for further analysis were dissected and homogenized in RLT buffer (Qiagen, Poland) or Trizol reagent (Invitrogen, Poland) using Ultra Turrax T8 homogenizer (Ika-Werke, Germany). Total cellular RNA from brains was isolated with RNeasy kits (Qiagen, Poland) according to the manufacturer’s instructions. The concentration and purity of RNA were determined by spectroscopy at 260/280 nm.

### Isolation of Mononuclear Cells from the CNS, Spleen and Lymph Nodes

After the anesthetization of animals and perfusion with ice cold PBS as described above, brains, spinal cords, spleens, axillary and inguinal draining lymph nodes were dissected and transferred into the ice cold PBS. Spinal cords were removed by intrathecal hydrostatic pressure using a syringe filled with ice cold PBS and a 20-gauge needle. Dissected brains and spinal cords were then immediately homogenized. This was done first by a thorough mechanical disruption using forceps and subsequently a disposable nylon mesh with 70 µm pores (BD Pharmingen). The homogenate was centrifuged for 10 min at 1500 rpm (4 °C). The pellet after careful mixing was resuspended in 40 % Percoll (Sigma-Aldrich, Poland) diluted with white Hank’s buffered salt solution (HBSS) (Sigma-Aldrich). The suspension of cells was then carefully overlayered on 70 % Percoll diluted with HBSS with phenol red (Sigma-Aldrich, Poland). The samples were subsequently centrifuged at 2000 rpm (4 °C) for 40 min with slow acceleration without the brake. Mononuclear cells forming a layer in the interphase between the red and white solution were then collected and homogenized in the TRIzol reagent (Invitrogen) to isolate the RNA. The protocol above was used for successful isolation of mononuclear cells from brains of animals with ChREAE. In case of naïve mice, however, even pooling brains and spinal cords from three animals was not sufficient to obtain a number of mononuclear cells enabling further studies.

In order to isolate mononuclear cells from the spleen and lymph nodes they were meshed trough a disposable sieve. Cells were later washed and pelleted by centrifugation (1500 rpm, 4 °C). Prior to the lysis of splenocytes the red blood cell (RBC) lysis buffer (NH_4_Cl, KHCO_3_ and Na_2_EDTA × 2H_2_O) was added to the pellet for 5 min on wet ice. After this step cells were pelleted again and homogenized with Trizol reagent.

### Isolation of PBMCs

Blood samples obtained by cardiac puncture of previously anesthetized animals were diluted with PBS and carefully layered onto Histopague 1077 (Sigma-Aldrich, Poland). The samples were then centrifuged at 1500 rpm (4 °C) with a slow acceleration and without the brake. Cells located in the interphase were collected, washed in PBS and pelleted. In the next step cells from the pellet were mixed and incubated with a RBC lysis buffer in order to lyse remaining red blood cells. Then PBMCs were resuspended in PBS, centrifuged and homogenized by vigorous pipetting in Trizol reagent (Invitrogen) for the isolation of total cellular RNA.

### Reverse Transcription Reaction and Real-Time PCR

The reverse transcription (RT) reaction was performed using 1 µg of total RNA, 10 mM dNTP mix (Applied Biosystems, Poland), 2.5 μM random nonamers (Sigma-Aldrich, Poland), 12.5 units of RNasin (Promega, Poland), 100 units of M-MLV and RT buffer (Promega, Poland). First, the 20 µl reaction mixture of RNA and nonamers was incubated for 5 min at 70 °C. Then the samples were cooled by placing them on the wet ice for one minute and the remaining reagents were added to each tube to a total volume of 50 µl. The RT reaction was performed at 37 °C for 50 min and then the samples were stored at −20 °C until needed.

For the analysis of expression of CCL19, CCL21, CCR7 and the histone (H3a) Rotor Gene 300 Apparatus (Corbett Research, Sydney, Australia) was used. Specific primers for murine CCL19 were as follows: Forward: 5′-CCTGGGAACATCGTGAAAG-3′, Reverse: 5′-AGCCCCTTAGTGTGGTGAACAC-3′, CCL21-Forward: 5′-AGGAAGAACCGGGAACCTC-3′, Reverse: 5′-AGGGCTGTGTCTGTTCAGTTCTC-3′, CCR7-Forward: 5′-CAAGAACCAAAAGCACAGCC-3′, Reverse: 5′-GACAAGGAGAGCCACCACC-3′ and H3A-Forward: 5′-AAGGAGGTGTCCTTACCATGGCTC-3′, Reverse: 5′-GCTTTTGTAGCCAGTTGTTTCTGG-3′. Each sample was run in duplicate and each experiment was performed three times. Two microliters of reverse transcription product for each sample was used as the template in a 20 μl reaction mixture containing Taq polimerase, PCR buffer, 25 mM MgCl_2_, 10 mM dNTPs (Applied Biosystems, Poland), Eva Green (Biomibo, Poland) and 10 µM specific primers (Institute of Biochemistry and Biophysics, Polish Academy of Sciences, Warsaw, Poland). QPCR Mouse Reference Total cellular RNA (Stratagene, La Jolla, CA, USA) was used as a positive control. Samples analyzed for expression of CCL19, CCL21 and CCR7 were run for 50 cycles with a hot start at 95 °C for 3 min and with steps of 95 °C for 30 s, 57 °C for 15 s, 72 °C for 30 s and 80 °C for 15 s. Samples analyzed for the expression of housekeeping H3A gene were run for 50 cycles with a hot start at 95 °C for 3 min and with steps of 95 °C for 30 s, 60 °C for 15 s, 72 °C for 30 s and 80 °C for 15 s. The amount of the analyzed message in each sample was calculated as proposed by Pfaffl (2001). The relative expression ratio (*R*) of an analyzed gene was calculated based on the efficiency (*E*) and the crossing point (CP) deviation of an unknown sample versus the control and expressed in comparison to the reference gene:

$${\text{Ratio}} = \frac{{(E_{\text{target}} )^{{\Delta {\text{CP}}_{{{\text{target}}({\text{control}} - {\text{sample}})}} }} }}{{(E_{\text{ref}} )^{{\Delta {\text{CP}}_{{{\text{ref}}({\text{control}} - {\text{sample}})}} }} }}.$$

### RNAse Protection Assay

The quantitative RNAse Protection Assay (RPA) was performed using custom made multiprobe template sets for selected analyzed chemokines and chemokine receptors (BD Biosciences, USA) as described before (Glabinski et al. 2003a). All procedures were done according to the protocol recommended by the manufacturer. In brief, [^32^P]-labeled RNA probes were synthesized and hybridized with 10 μg of analyzed RNA. Single-stranded RNA was then digested by RNase. The remaining protected double stranded probes were purified and resolved on denaturing polyacrylamide gel. The intensity of radioactivity for each gene was measured using PhosphorImager SI (Molecular Dynamics, Sunnyvale, CA, USA). The signal from each gene in every sample was quantitated and normalized to the signal from corresponding housekeeping genes (L32, glyceraldehyde 3-phosphate dehydrogenase). Two or three identical experiments were performed.

### Histology

Animals were deeply anesthetized with ketamine and transcardially perfused with 4 % formalin (Sigma-Aldrich, Poland). Organs of interest were removed, post-fixed for 2 days in 4 % formalin and embedded in paraffin. Series of sections (10 μm thick) were placed onto Superfrost plus slides (Super Frost Plus, Pittsburgh, USA).

### Antibodies and Immunostaining Protocol

#### Single Antigen Detection

Primary antibodies, polyclonal anti-CCL19 (catalog no. sc-9777) and anti-CCL21 (catalog no. sc-5811) were obtained from Santa Cruz Biotechnology (CA, USA), polyclonal anti-CD3 (catalog no. A045201) antibody was purchased from DAKO. We applied a standard protocol used in our laboratory for verification of antibody specificity. The protocol includes a series of negative controls with commercially available set of goat, rabbit and mouse immunoglobulins (Santa Cruz Biotechnology, CA, USA). Sections obtained from normal inguinal lymph nodes served as a positive control for verification of specificity of anti-CCL19 antibody (which labeled cells with morphology of either DCs and/or stromal cells) and anti-CCL-21 antibody (which labeled high endothelial venules). Additionally as usual on these occasions in order to verify any possible cross-reactivity of secondary antibodies we also labeled the tissue without any primary antibodies (data not shown).

For a single antigen staining (using anti-CCL19 or anti-CCL21 antibodies alone) the tissue was pretreated by microwaving with citrate buffer (2.1 g citric acid in 1000 ml of distilled water, pH 6) twice (3 min each with 5 min interval). Blocking of endogenous peroxidase activity was performed by incubation of slides with 0.3 % hydrogen peroxide in methanol. Before the application of primary antibodies the sections were incubated with a normal horse serum (Vector Laboratories, Peterborough, England) for 60 min. Diluted primary antibodies were put on sections and incubated overnight in a cold room. The secondary antibody and ABC kit used in subsequent steps were purchased from Vector Laboratories (Peterborough, England). 3,3′-Diaminobenzidine chromogen was purchased from Sigma-Aldrich (Poland). For a counterstaining we used cresyl violet (Sigma-Aldrich, Poland).

#### Triple Antigen Detection

For a triple antigen detection technique of immunofluorescence (immunofluorescence assay) was used. Secondary antibodies were combined with three different fluorochromes: Alexa Fluor^®^ 488 (green), Alexa Fluor^®^ 647 (far-red) and Cy3™ (red). For detection of CCL19 Ag we used anti-CCL19 antibody (see above) with donkey anti-goat Alexa Fluor^®^ 647 conjugated secondary antibody (Molecular Probes, Invitrogen France, cat. no 21447). For detection of CD3 antigen (see above for primary antibodies details) the secondary antibody was conjugated with Cy3™ fluorochrome (goat anti-rabbit IgG, Jackson ImmunoResearch, West Grove, PA, USA). For visualization of myelin basic protein (MBP) antigen a monoclonal anti-MBP (Chemicon Intl, cat. no MAB381) antibody with goat anti-mouse Alexa Fluor^®^ 488 conjugated (Molecular Probes, cat No A11001) was used. For each section we applied a retrieval method same as for single antigen detection (described above). All the primary antibodies were incubated overnight at 4 °C, secondary at room temperature for 60 min. The sequence of staining (in order to eliminate cross-reactivity) was as follows: overnight incubation with goat anti-CCL19 or CCL21 antibody in a cold room followed by incubation with donkey anti-goat secondary antibody. The next step was incubation with rabbit anti-CD3 antibody (overnight in a cold room) followed by detection using goat anti-rabbit secondary antibody. The final step was incubation with mouse anti-MBP antibody detected using goat anti-mouse secondary antibody. Incubation for 60 min with corresponding blocking sera preceded application of each primary antibody. For counterstaining of cell nuclei we used 4′,6-diamidino-2-phenylindole dichydrochloride reagent (Sigma-Aldrich, Poland) (not shown).

### Image Acquisition and Analysis

For analysis and image acquisition two different microscopes of Zeiss Inc. manufacture (Goettingen, Germany) were used. In both microscopes the following objectives made by Carl Zeiss Inc. were mounted: a plan: 5/0.12 and 10/0.25; LD plan-neofluar: 20/0.4; 40/0.6 and 63/0.7; EC plan-neofluar: 5/0.16, 10/0.3, 20/0.5 and 40/0.75: plan-apochromat: 63/1.4, 100/1.4.

For analysis and capture of bright field images we used an inverted Zeiss microscope type Axiovert 200. The images were acquired with a digital camera, AxioCam MRc5 (Carl Zeiss Group, Goettingen, Germany), attached to the microscope. Program Axio-Vision Rel. 4.2 (Carl Zeiss Group) served as the operating software for the camera. After acquisition the bright field images did not require further processing.

For analysis and image acquisition of sections stained with antibodies combined with fluorochromes we used a system for confocal imaging LSM 510 – Meta Confocor 2 with microscope Axiovert 200 M (Carl Zeiss Group, Goettingen, Germany). The immunolabelling visualized on the image (Fig. 3, panel c) in green color corresponds to the Alexa Fluor^®^ 488, yellow color to Alexa Fluor^®^ 647 and red color to fluorochrome Cy3™. The software used for image acquisition and analysis was LSM Image Browser (Carl Zeiss Group, Goettingen, Germany).

### Statistical Analysis

**Fig. 1:**
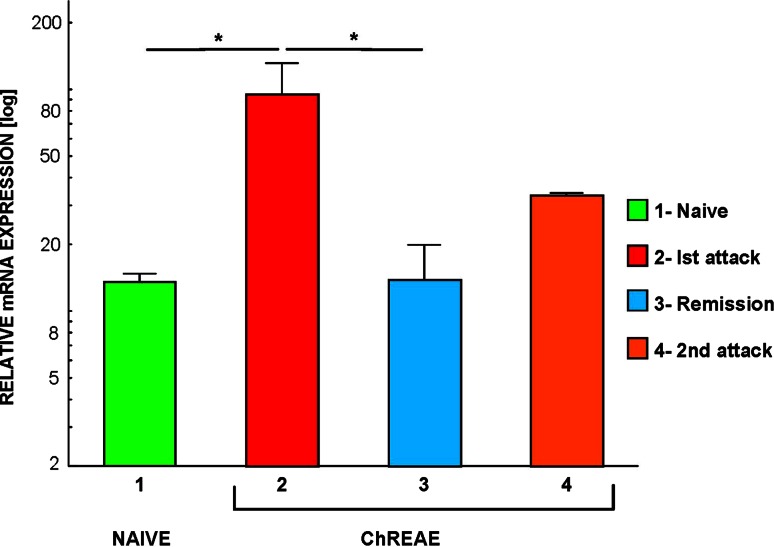
CCL19 mRNA expression in the CNS during ChREAE. The analysis of expression was performed using quantitative PCR (qPCR) technique in the whole brain homogenate during subsequent phases of ChREAE. *1* Naïve—nonimmunized control animals. *2*–*4* Groups of animals with ChREAE: first attack of ChREAE, remission and the second attack. Expression was analyzed by qPCR as described in “Materials and methods”. *n* = 5–8 animals were used in each group. Two identical experiments were done. Results from the representative experiment are presented on the graph. Significance is denoted as *p < 0.05

**Fig. 2:**
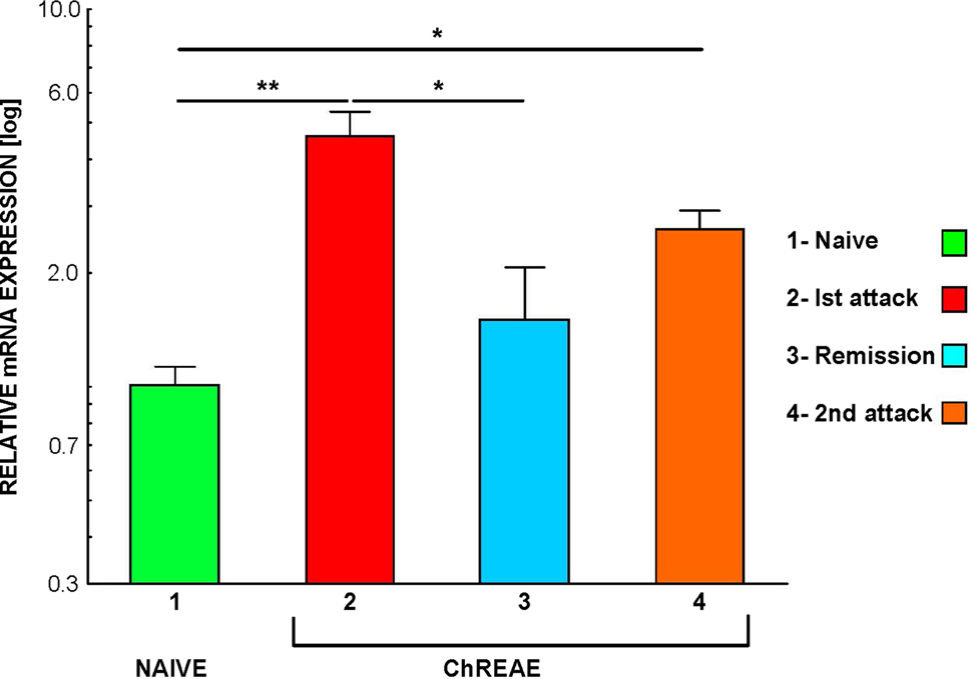
CCL21 mRNA expression in the CNS during ChREAE. The analysis of expression was performed using qPCR technique in the whole brain homogenates isolated during subsequent phases of ChREAE. *1* Naïve—nonimmunized control animals. *2*–*4* Groups of animals with ChREAE: first attack of ChREAE, remission and with the second attack. Expression was analyzed by qPCR as described in “Materials and methods”. *n* = 5–8 animals per group. Two identical experiments were done and results from the representative experiment are shown. Significance is denoted as *p < 0.05; **p ≤ 0.01

For statistical analysis a nonparametric Mann–Whitney *U*-test and a Kruskal–Wallis test were applied. In order to correct for multiple comparisons the false discovery rate method was used. *p* values below 0.05 were considered significant. In order to indicate a statistical significance on the figures we used a convention as follows: (*) *p* ≤ 0.05, (**) *p* ≤ 0.01, (***) *p* ≤ 0.001. The data presented on Figs. 1, 2, 4, 5a, b, are presented as mean + SEM or ±SEM.

## Results

### CCL19 Expression Increases During Active ChREAE

We observed a statistically significant difference of expression of CCL19 between groups: healthy control, mice with the first attack, remission, and the second attack of ChREAE (*p* < 0.05) (Fig. 1). Further analysis showed that the expression of CCL19 in the CNS was upregulated in brain homogenates during the first attack of the disease (*p* = 0.02) (Fig. 1). During remission of ChREAE, the expression of CCL19 was significantly lower than during the first attack of the disease (*p* = 0.02) (Fig. 1). Although we observed an increase of CCL19 expression during second attack of ChREAE it did not reach statistical significance. The expression of CCL19 in brain and spinal cord homogenates analyzed using the RPA technique showed a similar pattern (data not shown).

### Expression of CCL21 is Increased in CNS During Active ChREAE

The analysis of expression of CCL21 in the CNS homogenates isolated from healthy animals, mice with the first attack, remission and the second attack of ChREAE showed a statistical difference between these groups (*p* = 0.003) (Fig. 2). The expression of CCL21 was upregulated in brain homogenates during the first and the second attack of ChREAE (*p* = 0.008 and 0.02, respectively) (Fig. 2). CCL21 expression during remission was significantly lower than in the first attack (*p* = 0.02) (Fig. 2). The expression of CCL21 in the brain and spinal cord homogenates analyzed using the RPA method showed a similar pattern (data not shown).

### Localization of CCL19 and CCL21 Expression in the Brain During ChREAE

**Fig. 3:**
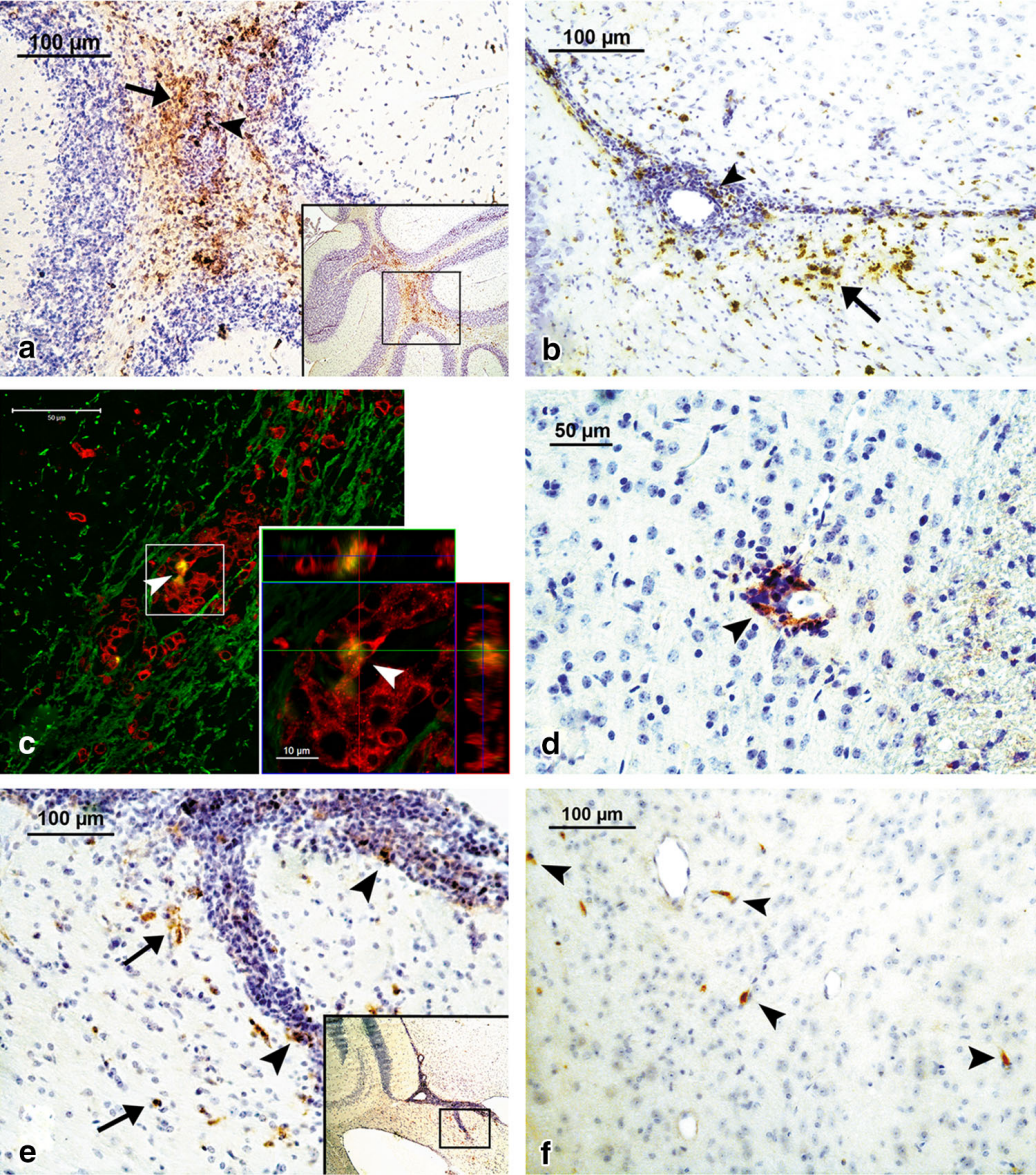
Immunohistochemical analysis of expression of CCL19 (panels **a**, **b**, **d**), CCL21 (panels **e**, **f**) and colocalization of CCL19, CD3 and MBP antigens (panel **c**) in the brain during ChREAE. **a** CCL19 is expressed abundantly during the first attack of ChREAE in many areas of the brain including the cerebellum (*inset*). CCL19-positive cells, most of them displaying a morphology of mononuclear leukocytes are present within perivascular inflammatory cuffs (*arrowhead*). A strong staining is also present in surrounding infiltrated parenchyma (*arrow*). **b** During the first attack of disease the expression of CCL19 was detected also in the infiltrated periventricular white matter in the vicinity of inflammatory lesions (*arrows*). In these inflammatory lesions expression of CCL19 was also present (*arrowhead*). **c** CCL19 (*yellow*) and CD3 (*red*) are expressed in the same inflammatory lesion in the periventricular white matter (myelin basic protein, MBP—*green*). A high magnification of this lesion shows (*arrowheads*) that expression of CCL19 (*yellow*) is colocalized with some cells expressing CD3 (*red*). **d** During chronic phase of the disease expression CCL19 in the brain was also detected using immunohistochemistry but in much lesser degree near only a small number of inflammatory lesions. **e** CCL21 expression was observed during the first attack in many areas of the brain also near the lateral ventricles (*inset*). Higher magnification shows that positive staining was present both within (*arrowheads*) and near (*arrows*) inflammatory cuffs, in the infiltrated brain parenchyma. **f** During the chronic phase of the disease CCL21 staining still could be detected but only few positive cells were observed in brain parenchyma in the vicinity of blood vessels (*arrowheads*). The immunostaining procedure was performed according to the protocols described in “Materials and methods”

During the active phase (the first attack) of ChREAE, CCL19 was abundantly detected by immunohistochemistry in mononuclear cells showing a leukocyte morphology and localized within inflammatory foci and in surrounding brain parenchyma in line with PCR results (Fig. 3, panels a–c). Brain infiltrating cells in the areas of CCL19 expression formed typical perivascular cuffs or were localized in submeningeal regions. As described before, cells with a macrophage morphology showed colocalization with CCL19 staining (Fig. 3, panel a and b). Some endothelial cells were also CCL19-positive (data not shown). Using a triple antigen staining protocol and confocal microscopy we observed that at the early stage of the disease (day 1) abundant CD3^+^ cells (red) were present within inflammatory lesions (Fig. 3). CCL19-positive labelling was observed in the same areas (Fig. 3, arrowhead). At this stage of the disease only minor damage to adjacent myelin (green) in these sites of infiltration was observed. Further analysis of high magnification images showed colocalization of CCL19 and a certain number of cells with CD3-positive labelling within perivascular cuffs (Fig. 3c, inset-arrowhead). The chronic phase of the disease was characterized by the presence of only a small number of CCL19^+^ cells localized near small perivascular infiltrates (Fig. 3d, arrowhead). CCL21-positive cells in the acute stage of our model were also present in the areas of inflammation (Fig. 3 panel e, inset). The positive staining, similarly to CCL19, was confined almost exclusively to cells present within perivascular cuffs and those in close vicinity to inflammatory lesions (Fig. 3e). However, some endothelial brain vessels were labeled (data not shown). The CCL21 expression during the chronic phase was much less intense and limited to only a few dispersed cells in the vicinity of the blood vessels (Fig. 3 panel f, arrowheads).

### Expression of CCL19 and CCL21 Increases During Active ChREAE in Mononuclear Cells Isolated from Blood, and from the CNS but Not in Lymph Nodes and Spleen

**Fig. 4:**
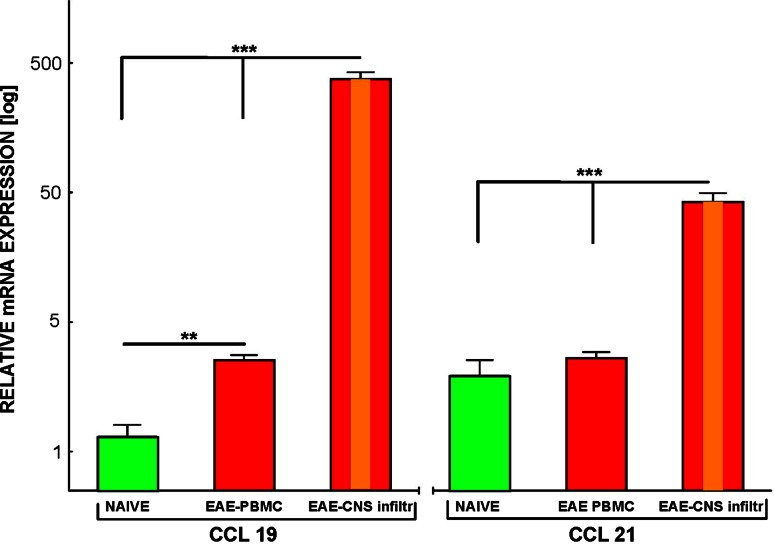
Expression of CCL19 and CCL21 in PBMC and mononuclear inflammatory cells isolated from the CNS (CNS infiltr) during ChREAE. Analysis of CCL19 and CCL21 expression in PBMC was performed in normal control (NAIVE) and during the first attack of ChREAE (EAE-PBMC, EAE-PBMC) using qPCR. Isolation of PBMC and mononuclear cells from the CNS is described in detail in “Materials and methods”. In each group 5–12 animals were used. Healthy (naïve) animals were used as a control. Two identical experiments were done. Results from the representative experiment are shown. Significance is denoted as **p ≤ 0.01; ***p ≤ 0.001

There was a statistically significant difference between the expression of CCL19 and CCL21 in mononuclear cells between the samples obtained from the blood of normal animals, blood from animals with ChREAE and infiltrating mononuclear cells isolated from the CNS during ChREAE (*p* < 0.001). The expression of CCL19 in PBMCs significantly increased during the first attack of ChREAE (*p* = 0.003) in comparison to normal controls (Fig. 4). In the mononuclear cells obtained from CNS parenchyma isolated during the first attack of ChREAE, increased of expression of CCL19 was much more evident (approximately 100-fold). Measured expression of CCL19 in this group was significantly higher in comparison not only to PBMCs of healthy controls but also to PBMCs obtained from animals with ChREAE (*p* < 0.001). In contrast to CCL19, the expression of CCL21 in PBMC obtained from animals with the first attack of ChREAE was not upregulated (Fig. 4), however, in the mononuclear cells isolated from the CNS at that time we observed a significant increase of CCL21 expression in comparison to normal controls (*p* < 0.001). Mononuclears isolated from peripheral lymphatic organs were obtained by draining lymph nodes and spleen during the first attack of ChREAE, expression of CCL19 and CCL21 was then measured by a quantitative PCR and found similar to the respective expression observed in control animals (data not shown). The analysis of expression using the RPA method showed a similar pattern (data not shown).

### CCR7 Expression is Decreased in PBMC but not in Lymph Node Cells and Splenocytes During Active ChREAE

**Fig. 5:**
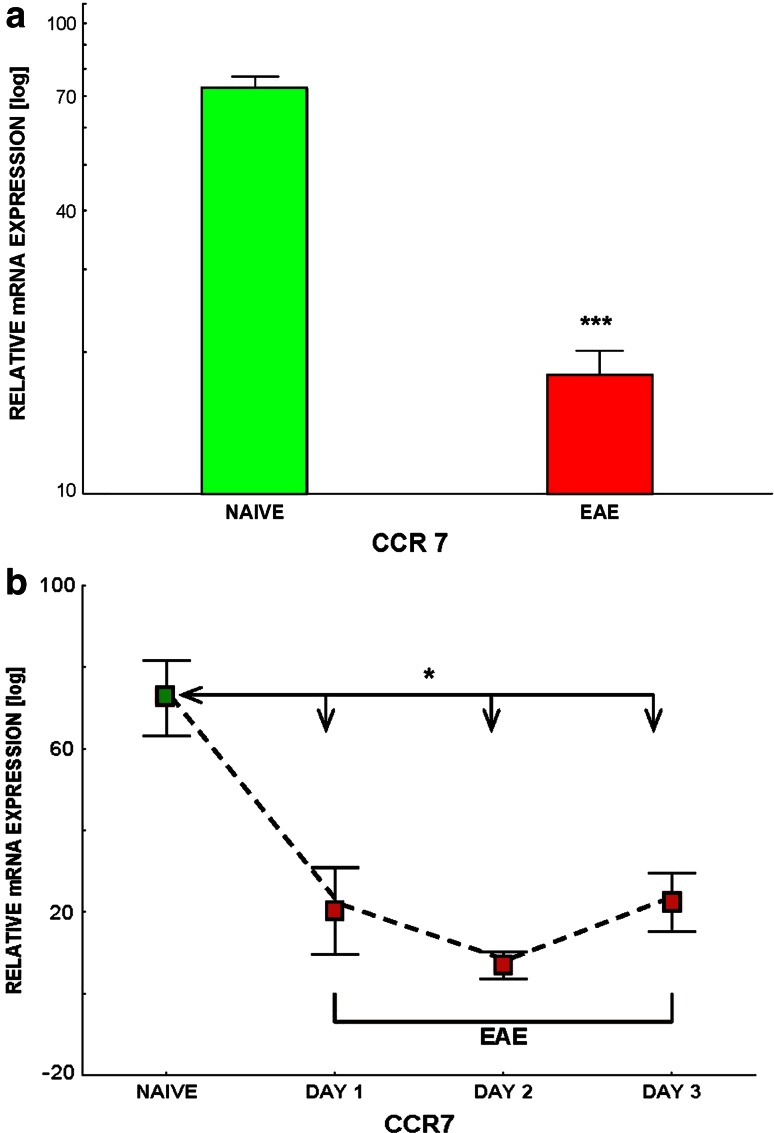
**a**, **b** Comparison of expression (analysis using qPCR) of CCR7 in mononuclear cells isolated from the blood of control animals (NAÏVE) and in animals with acute phase of ChREAE (EAE). The isolation method of PBMC is presented in “Materials and methods”. Each *box* represents the group of 5–12 animals. The representative experiment is shown. **b** The kinetics of expression of CCR7 by PBMC during subsequent days following development of the first clinical symptoms of ChREAE. Significance is denoted as *p < 0.05

A statistically significant decrease in CCR7 expression in PBMC was observed during the first attack of ChREAE (*p* = 0.001) (Fig. 5a). The analysis of the kinetics of CCR7 expression in PBMC showed a rapid and significant decrease during the first 3 days of the disease (*p* = 0.025, 0.025 and 0.013, respectively) (Fig. 5b). The expression of CCR7 in lymph node cells and splenocytes was not changed during ChREAE in comparison to normal animals (not shown).

## Discussion

CCL19 and CCL21 belong to the group of constitutively expressed lymphoid (homeostatic) chemokines. They play an important role in control of migration of T-cells and DCs to secondary lymphoid organs (Forster et al. 2008). Recently the role of CCL19 and CCL21 during the development of diseases has also gained much interest. It has been shown that CCL19 is constitutively expressed in the CNS and its expression significantly increases during MS in active as well as chronic inflammatory brain lesions (Krumbholz et al. 2007). Experimental studies on acute EAE, animal model of MS further indicated that CCL19 and CCL21 are expressed during the initial phase of the disease and play an important role in the development of autoimmune neuroinflammation (Alt et al. 2002). One of previous studies showed also that the source of CCL19 in EAE brains were cells present in perivascular inflammatory foci, including infiltrating leukocytes as well as some astrocytes and microglial cells, while CCL21-labelling was associated with the vessels around inflammatory foci (Columba-Cabezas et al. 2003). The pathology of EAE is known to be driven by IL-17A-secreting Th17 cells and IFN-γ-secreting Th1 cells (Kroenke et al. 2008). Recent experimental studies show that CCL19 and CCL21 through development Th17-dependent autoimmune responses are important for development of EAE (Comerford et al. 2010; Kuwabara et al. 2009, 2012). In the present study we analyzed the expression of both chemokines in the CNS, and in peripheral tissues including blood, spleen and lymph nodes during chronic-relapsing EAE (ChREAE). The ChREAE model closely resembles the most frequent form of MS, relapsing-remitting MS. This experimental approach permitted us to accurately gauge the involvement of CCL19 and CCL21 in inflammatory cell trafficking during autoimmune neuroinflammation in the CNS. In the first part of the study we have shown in the CNS an important increase of expression of CCL19 during first attack of ChREAE and of CCL21 during first and second attack of the disease. Further analysis showed that during the initial stage of ChREAE an important increase of expression of CCL19, but not CCL21, in PBMCs. Interestingly, in CNS-infiltrating mononuclear cells isolated from brains and spinal cords, the level of expression of CCL19 was nearly 100-fold higher, and CCL21 almost 10-fold higher, than in PBMC. This observation supported those data suggesting that at the initial stages the main source of CCL19 and CCL21 in the autoimmune CNS inflammation are inflammatory cells accumulating in the CNS, with PBMC as an important additional source of CCL19. One of the possible interpretations of these data is that inflammatory cells migrating from the blood to the CNS first upregulate CCL19 expression after exiting the lymph nodes and further increase this expression after passing the blood–brain barrier (BBB). In the case of CCL21, cells upregulate its expression only after passing the BBB. We did not find any difference between the expression of CCL19 and CCL21 in mononuclear cells isolated from lymph nodes and spleens of naïve control and animals with ChREAE at any stage of the disease (not shown).

We further studied the cellular source of CCL19 and CCL21 in the CNS compartment during ChREAE using immunohistochemistry. We confirmed that during the first attack of ChREAE many CCL19^+^ cells have a typical morphology of activated macrophages/microglia, but we also observed a colocalization of CCL19^+^ labelling with a certain number CD3^+^ T-cells. This finding extends previous observations showing the presence of CCL19^+^ cells, although few in number, in areas containing mainly T-cells (Columba-Cabezas et al. 2003). This also indicates that an as yet unidentified subpopulation of T-cells, in addition to macrophages and DCs, could also be the source of CCL19 at a certain time point in the acute phase of ChREAE. Further analysis is required to precisely determine the subpopulation of T-cells that might be the source of this chemokine.

CCR7 is a common receptor for CCL19 and CCL21 and it is expressed on naïve T-cells and on many subpopulations of immunocompetent cells (Debes et al. 2004; Ohl et al. 2004; Parlato et al. 2001). The CCR7 has been identified as a stimulator of migration of regulatory T-cells expressing CD4, CD25 and forkhead box p3^+^ (FoxP3), known as regulatory T-cells, which play a role in controlling of inflammatory responses (Schneider et al. 2007; Ueha et al. 2007). It has been also shown that CCR7 expression is upregulated in the CNS during both MS and EAE, mostly by activated microglia and DCs (Alt et al. 2002; Columba-Cabezas et al. 2003; Kivisakk et al. 2004; Serafini et al. 2006).

We observed previously that the expression of CCR7 by inflammatory cells accumulating in the CNS increases during attacks of ChREAE (Bielecki et al. 2007). In the present work we found for the first time that the expression of CCR7 on PMBC undergoes a significant and rapid decrease during the acute phase of ChREAE. Interestingly, we did not observe diminished expression of CCR7 in immunocompetent cells isolated from lymph nodes and spleen at that time (not shown). We can only speculate that the cells which do not cross the BBB downregulate the receptor upon exposure to CCL19 (Britschgi et al. 2008). Another possibility is that the disease induction may cause the reduction in the central memory CD4^+^, CCR7^+^ T-cells in the blood. The results presented here are in line with a hypothesis that during early EAE, chemokines either amplify or modulate the ongoing inflammatory process, most likely initiated by a specific immunological recognition of myelin autoantigens by activated immunocompetent cells (Glabinski et al. 2003a).

In the present study we demonstrated for the first time that the expression of CCL19 but not CCL21 is upregulated simultaneously in immunocompetent cells in the blood and in the CNS during ChREAE. Additionally we have shown for the first time a subpopulation of CD3^+^ cells expressing CCL19 present within inflammatory lesions without any evident signs of demyelination during the acute phase of ChREAE. Moreover, we found that the expression of CCR7, the receptor for CCL19 and CCL21, is decreasing during the early stage of ChREAE in the PBMC. We believe that our observations provide valuable information about the mechanism of development of ChREAE and are an important addition to a very complex picture describing mechanisms leading to autoimmune CNS inflammation. CCL19 and CCL21, as well as their common receptor the CCR7, appear to be important factors not only in the initiation of the CNS pathology during the early stage of MS, but also in the regulation of the inflammatory response, and seem to be interesting targets for future therapeutic interventions.
